# Simulation as a pedagogical learning method for critical paediatric nursing in Bachelor of Nursing programmes: a qualitative study

**DOI:** 10.1186/s41077-020-00140-2

**Published:** 2020-09-03

**Authors:** Iben Akselbo, Heidi Killingberg, Ingvild Aune

**Affiliations:** Department of Public Health and Nursing, Faculty of Medicine and Health Sciences, NTNU (Norwegian University of Science and Technology), 7004 Trondheim, Norway

**Keywords:** Simulation, Nurse student, Paediatric emergency, Learning, qualitative study

## Abstract

**Background:**

In the national education plan for Bachelor of Nursing in Norway, it is emphasized that focus areas for practical studies must include experience related to paediatric nursing. However, given the paucity of children’s wards in Norwegian hospitals, few students are offered this. The purpose of this study is to explore undergraduate nursing student’s perception of using simulation as a learning method to obtain knowledge and skills in delivering quality healthcare to children and their parents in emergencies.

**Method:**

A total of 36 students participated in focus groups. The students were asked to reflect on their learning outcomes regarding the educational method during the simulation. In addition, the students were encouraged to discuss whether this pedagogical method was useful in preparing them to deal with critical medical situations in relation to children and their parents. The interviews were transcribed and analyzed using qualitative content analysis.

**Results:**

Three subjects emerged from the analysis. The first, simulation as an educational method, showed that the students thought that simulation gave a greater degree of realism and seriousness than other learning activities. The second subject, preparedness for later practice, showed that the students perceived simulation as one of the ways in which they were best prepared for the profession as a nurse. The students emphasized the benefit of having concentrated on children and their parents. The third subject, stress and leadership, showed that simulation as a method was stressful to most students, and lack of knowledge and anxiety about conducting other students kept them from taking the lead. However, the students experienced that they learned a lot about themselves and how they appear as nurses.

**Conclusion:**

The bachelor-level student nurses experienced simulation as a realistic and effective educational method for gaining knowledge in the critical healthcare of children and their parents. Simulation made them reflect on a nurse’s area of responsibility in emergencies. When offered practical experience in children’s wards, the use of simulation as a didactic method may help students develop sufficient competence to act appropriately and expediently in critical paediatric nursing settings.

**Trial registration:**

The study (number 52776) was approved by the Norwegian Centre for Research Data.

## Background

In the national education plan for Bachelor of Nursing programmes in Norway, practical studies must include experience in paediatric nursing [1]. However, given the paucity of children’s wards in Norwegian hospitals, few students are offered this clinical experience. Furthermore, demands and expectations for healthcare professionals to provide safe and secure services are increasing. Since 2011, the Ministry of Health and Care Services has aimed to reduce unnecessary patient injuries in health services and contribute to the building of lasting systems and infrastructure for patient safety, as well as to improve patient safety culture [2].

To meet the requirements of the education plan, a university in Norway conducted a study using health-related simulation of acute nursing for children as part of theoretical studies in the second year of the Bachelor of Nursing programme. Project participation was expected to increase knowledge of nursing for children and young people. The learning outcomes from the study plan that were considered relevant included student knowledge of (a) nursing for patients with acute, critical, and chronic illness and suffering; and (b) communication and interaction with a focus on acute, critical, and chronically ill patients and their relatives [3].

To strengthen the competence in nursing for sick children and their parents, simulation as an educational method can be beneficial. The use of simulation has shown to increase students’ ability to think critically [4]. Simulation has been utilised increasingly often as a teaching strategy in nursing education programmes. Pamela Jeffries defined *simulation* as ‘activities that mimic the reality of a clinical environment and are designed to demonstrate procedures, decision making, and critical thinking through techniques such as role-playing and the use of devices such as interactive videos or mannequins’ [5]. Communication and interaction are important factors in nursing practice; therefore, simulation can be useful in improving this practice. The more often students participate in simulation, the greater their potential progress [6]. Practice periods can be replaced with simulation to a greater or lesser extent, and yet provide the same learning outcomes in relation to skills and knowledge [7].

Simulation is considered a safe method when learning to cope with unforeseen situations in relation to non-technical skills, thereby improving management abilities [8]. However, the acquisition and maintenance of equipment and audio-visual solutions for high-fidelity simulation is more expensive than other teaching methods. Teachers must also devote substantial time to planning meetings, creating and conducting scenarios, implementing challenging cases, finding necessary equipment, cleaning up, and evaluating student performances [9, 10]. In addition, staff must be adequately trained and continuously updated on equipment, method, and scenarios [11].

Learning through experience is a crucial element of the simulation process. Learning theories, such as Kolb’s experiential learning theory [12] and Schön’s reflection theory [13], are based on experiential learning. According to Kolb’s theory, learning occurs through change and adaptation of what one already knows. Adaptation and reflection are tools used to reach a new recognition in action [12]. Kolb suggested that learning is an ongoing, cyclical process, consisting of four stages. The process begins with the concrete experience of a situation. This is followed by reflective observation, during which the student views the experience from as many perspectives as possible and reflects on its significance. The insight established through these reflective observations becomes the basis for abstract conceptualisation as the student attempts to organise and generalise experiences and abstract them into new theories and hypotheses. These become, in turn, the basis for active testing and experimentation. In this stage, the student is asked how to act the next time he or she encounters a similar situation and how he or she will apply the new knowledge. At this point, the student has learned how to make decisions and act when encountering a similar situation in the future. These new experiences then form the foundation of the next stage of the learning cycle, which recommences with the first stage.

Schön introduced the concept of the ‘reflective practitioner’ [13]. This represents a further development of the action aspect of Kolb’s learning cycle. Here, Schön distinguished between the reflection that occurs while action is being taken and the critical reflection that occurs after an action has been performed. Critical reflection on the chosen solution involves a discussion of how the action can be justified by theory and practice. Such reflection will lead to an awareness of why a chosen decision was preferred over others [14].

In 2013, the World Health Organization (WHO) stated that health education institutions should use simulation in the education of health professionals [15]. The WHO further underlined in their 2018 report *Simulation in nursing and midwifery education* that ‘evidence from multiple studies shows that simulation is a highly valuable strategy for training nurses and midwifes’ [16]. Randomised controlled studies also support the use of simulation in the preparation of graduate nursing students [17, 18].

Nurse educators experience difficulties providing undergraduate students with paediatric learning experiences [19]. Students are exposed to difficult situations during simulation, which they may not have encountered in the clinical arena [20]. A reduced number of placements, inconsistency in the quality and availability of learning experiences, and increased parental collaboration in the caregiving process of sick children have made learning in clinical studies more challenging [21]. Simulation has the potential to complement learning in clinical and classroom settings and may therefore develop the essential skills required for the students [19, 20, 22, 23]. The aim of this study was to explore undergraduate nursing student’s perception of using simulation as a learning method to acquire skills in quality healthcare to children and their parents in emergencies.

### Preparing for simulation

Preparing the students and the environment is a basic condition for successful simulation. According to the International Nursing Association for Clinical Simulation and Learning [24], all simulation-based experiences begin with the development of measurable objectives designed to achieve expected objectives. Therefore, a programme was developed with a description of scenarios with expected learning objectives, which was published on an e-learning platform for the 36 nursing students involved. These scenarios were validated by practising paediatric nurses. The students were introduced to the simulation topic 2 days before the simulation took place by viewing a film about children’s experiences of being hospitalised. At this time, the students were divided into six groups of six students each. Each group was assigned different themes to work with throughout the week.

Class lectures informed the students about the simulation process by reviewing the phases of the method. The students had one simulation training in the first year of their study, where they simulated in large groups. Students were assured that the simulation would not be filed or registered for future use and that any mistakes or failures regarding relevant actions during the session would not be used for formal evaluation. The students were informed that the scenarios were to be videotaped and used during the debriefing and then deleted.

To ensure simulation quality (including realism and relevance), the instructors in charge of such a method must have substantial expertise [25]. Therefore, the staff conducting the simulations included two assistant professors educated as facilitators in simulation and with backgrounds as experienced paediatric nurses. One of the facilitators was responsible for briefing and debriefing the students, as well as observing the scenario, whereas the other was in charge of videotaping and streaming to the observer unit, conducting the simulators and scope, and was the voice of both the doctor on the phone and the 6-year-old patient. A third assistant professor with facilitator competence assumed the role of the concerned mother of the child.

**Fig. 1:**
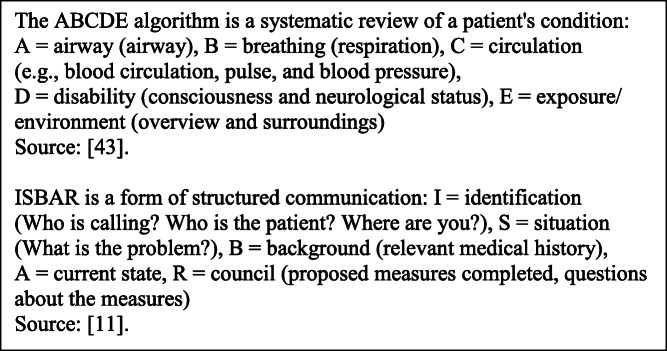
The ABCDE algorithm and ISBAR

The descriptions of learning objectives in education programmes are often quite abstract and therefore difficult to evaluate concretely [26]. Based on this, the learning objectives were designed to be understandable to the students with clear and measurable expectations:

- Students will be able to use ABCDE (Fig. 1) and act in consideration of the situation; and
- Students will be able to interact, conduct, and communicate in emergencies using ISBAR (Fig. 1, [27]).

Students who were respondents received tasks in relation to these objectives.

### The briefing

Students received a 30-min briefing, during which the environment of the simulation was presented. A physical learning environment was created that included a hospital bed, oxygen, suction systems, and monitoring equipment to measure blood pressure, pulse, and respiration. The SimJunior 2015® (Laerdal Medical) simulator with respiration movements, respiration and heartbeat sounds, and palpable pulse was used in the first scenario, and SimNewB 2016® (Laerdal Medical) with the ability to show circumoral cyanosis, palpable pulse, heartbeat, and respiration sounds was used in the second scenario. The students were instructed to ask the facilitator for information about skin colour, capillary refill, and temperature. A mobile phone was available for calling the doctor (the operator), and there was a table that displayed different relevant equipment, such as saline for infusion, 1 mg/ml of adrenaline, syringes and needles, disinfectant, gloves, an inhalation chamber for children, and stethoscopes. The facilitator pointed out the placement of the cameras and reviewed the learning objectives with the students. The group was then divided into two separate units of three students: one respondent unit and one simulation unit. The groups switched roles between scenarios, and each scenario lasted for approximately 15 min. Prior to beginning the simulation, the operator provided brief instructions to the respondents (now placed in a room for streaming of the scenario) on how to give positive feedback to their fellow students.

### Description of scenarios

#### Scenario 1 (see Additional file 1)

Boy, 6 years old and 25 kg, is at the hospital with his mother. The child came to the hospital early this morning, and his first intravenous antibiotics were administered 8 h ago. The students, acting as nurses, have just arrived on shift, and the previous nurse started the intravenous antibiotics before she left. The mother calls for help. When assessed, the boy is slack and has cyanosis. His oxygen saturation is 78%, pulse is 115, respiration rate is 55 with stridor, and blood pressure is 75/40. The capillary refill is 4–5 s, and he is itching due to urticaria. The mother seems afraid and worried.

#### Scenario 2 (see Additional file 2)

Girl, 5 weeks old and 5 kg, has come to the children’s emergency at the hospital after having a cold for 3 days and experiencing problems with breastfeeding. Her temperature is 38.5 °C. Diagnosis is respiratory syncytial virus.

Sodium chloride inhalation has been administered during the emergency, and now the child has been transferred to the children’s infectious diseases ward. The child’s cardiac function is being monitored. The students, acting as nurses, have just arrived on shift. The mother calls for help, and one of the students answers the call. The child is experiencing respiratory distress with stridor, a respiration rate of 65, and cyanosis. Oxygen saturation is at 72%, pulse is 160, capillary refill 4 s, and temperature is 38.5 °C. The mother seems desperate and afraid. The student calls the other students for help.

### Debriefing

The model used was the ‘diamond debriefing’ method which consists of three phases: description, analysis, and application [28]. It lasted for approximately 45 min and took place in a classroom after the first scenario. It was then repeated after the second scenario when the students had changed roles. Debriefing began with the description phase in which the students had to describe what they had accomplished in the scenario. Next, the students were asked whether anything should have been done differently. The rationale for interventions was highlighted, and alternative options were discussed. In the application phase, the students had the opportunity to evaluate their skills, and the facilitator gave constructive feedback to each of them. The respondents were invited into the debriefing session, and their observations, as well as achievement of learning objectives, were discussed. Video of the simulation was used to emphasize important situations.

## Method

### Data collection

Following the simulations, the 36 students were invited to participate voluntarily in focus groups (six students in each group). Focus groups are particularly well suited to learning about experiences, attitudes, or views in an environment in which people interact [29]. A focus group makes it easier to stimulate the collective memory and raise awareness of a common knowledge base that may initially seem trivial and unimportant to the individual [30]. At the university, a professor who was unknown to the students and who had no involvement in the bachelor’s programme conducted six focus groups, retaining the original student groups used during the simulation. All of the students involved in the simulations consented to their participation in the focus groups. All of the students were women between the ages of 20 and 30. The study (number 52776) was approved by the Norwegian Centre for Research Data. The focus groups took place 1 week after the simulation and lasted approximately 1 h each. They took place in a group room and were tape-recorded. During the focus groups, the students were asked to reflect on the learning outcomes of the educational method in terms of their own and the groups’ communication and actions in the simulation’s critical situation (see Table 1 for the interview guide). In addition, the students were encouraged to discuss whether this pedagogical method had been useful in preparing them to appropriately respond to critical paediatric situations involving children and their parents.

**Table 1:** Interview guide

1	What are your general experiences with simulation as an educational method?
2	Reflect on the use of video recording and the practice of leadership in emergency situations.
3	How do you feel simulation as a learning method compared to other learning methods when it comes to paediatric emergency?
4	Reflect on the value of simulation during your education in relation to your role as a future nurse.

### Analysis

The recorded focus groups were first transcribed by the two assistant professors who conducted the simulation. During the analysis, the same two assistant professors created codes independently and then came together and reached consensus. The analysis of the data was conducted using qualitative content analysis [31]. The first step in the data analysis was to read transcriptions of all six focus groups to obtain a general sense of all relayed information. Next, the focus groups were read word-for-word to derive codes by highlighting the text that featured key thoughts or concepts. From this, labels for codes emerged that were reflective of more than one key thought, and codes were then sorted into categories based on how the different codes were related. These categories were used to group codes into meaningful clusters, and definitions for each category were then developed. Examples from the analysis process are shown in Table 2.

**Table 2:** Example from the analysis process

Meaning units	Code:	Category
It was useful to practice, as you become familiar with how to act in an emergency	Realistic learning environment	Preparedness for practice

## Results

Three categories emerged from the analysis: simulation as an educational method, preparedness for later practice, and stress and leadership.

### Simulation as an educational method

The students expressed that simulation provides a higher degree of realism and seriousness than skill training. They indicated that, during skill training, they were more susceptible to fooling around, but that the simulations more accurately portrayed the gravity of the situation and thus captured their full and immediate attention. This was perceived as very beneficial to the learning process. The students were motivated by the fact that there was a patient who was sick. ‘It was like a riddle to solve’ (FG1 P5—for focus group 1, participant 5). The simulation was also described as more serious than skill training because of the videotaping and knowing that others were watching. Another student explained:

During skill training you are more left to yourself and can more easily slip out in relation to the task (FG3 P2).

The students argued that they had learned more from a simulation followed by debriefing than they would from an entire day of lectures. One student suggested that teaching respiration and circulation would have been more effective if simulation had been used as a teaching method: ‘I think it’s easier when I can relate to a situation. Then I can think back to what happened then, instead of just sitting and looking at a PowerPoint or in a book’ (FG3 P4).

The students also expressed that they had learned something new about themselves in terms of how they behave and deal with situations (e.g. in relation to stress and communication). As the simulation took place in a safe environment, making mistakes was not a matter of life or death. This sense of security gave the students the opportunity to practice and learn from their mistakes before applying their skills to real-life emergencies. In addition, they felt that they could rely on their teacher during crucial decision-making moments and that this would not be used against them. However, many commented on the importance of conducting simulations in small groups. Several of the students found the video recording uncomfortable, but also recognised the learning outcomes. The students thought simulation could be used more often to gain confidence in applying their skills. In contrast, some students reported that they felt the situation was artificial and that they had difficulty immersing themselves into a situation involving a mannequin rather than a real patient. As described by one student:

I don’t manage to think that it’s a real situation then. I don’t think I had acted equally if there was a real person lying there (FG4 P1).

Debriefing, involving reflection and feedback from both the teacher and other students, was seen as very important. As stated by one student:

[Debriefing was helpful to] get through the situation, get your mind calm before embarking on something else. And getting others’ views on the situation (FG6 P3).

The students also identified the teacher’s competence in simulation as important. When the teachers emphasised what a student had done well and gave suggestions on what could have been done differently, it gave the student a feeling of mastery. The students noted the quality of the structure and logistics during the 3-h simulation and the importance of these factors in helping them maintain their concentration.

Nevertheless, students mentioned that participation in the simulation might be intimidating if the students knew the teachers or thought they might meet them again later on in their education (e.g. in practice for evaluation). One student discussed this as a worry:

If I had made a really big mistake on the simulation, then I would have felt that she had remembered it, even though she had not said it (FG5 P2).

Furthermore, the students pointed out that the learning objectives could also be limiting because many other things happened along the way:

You learn much more than the stated learning objects, anyway (FG2 P2).

### Preparedness for later practice

Aside from practice, simulation was perceived by the students to be one of the most effective ways of preparing themselves for the profession of nursing. The students acknowledged the usefulness of acting out an emergency situation, noting that it helped them to feel that their body was equipped to cope with stress and that they knew what to do. While not all students experienced stress, those who remained calm could see that others around them had lost control. They found it useful to feel other students’ stress and practise what they could do to calm the situation. Using the ABCDE algorithm to prioritise actions provided the students with a sense of confidence. Many students found that they learned better and remembered more using the ABCDE algorithm than with other methods:

You remember better, it is a bit limited what you remember from the curriculum books all the time. Especially a book that has a lot of text. So, it’s kind of … such situations are remembered better later (FG3 P6).

The students emphasised the benefit of having concentrated on measuring vitals in children and acting accordingly, while also striving to relate to and calm children of different ages. Several students experienced the mother in the scenario as disruptive to the situation and felt it was difficult to concentrate when she asked questions along the way. At the same time, they found it interesting to deal with an emotional mother who wants to ensure her child’s well-being. The students noted that giving the mother a task—such as holding her child’s hand, holding the oxygen mask, or keeping her baby on her lap—calmed her down in the situation. The students found it particularly valuable to acquire this knowledge before requiring it in practice:

And that’s the thing especially in paediatrics, that relatives are so important. You may not say “please calm down” but preferably two metres away from the child (FG1 P5).

### Stress and leadership

Prior to beginning the simulation, several of the students were uncertain and nervous. They noted that they expended a lot of energy due to the physical and mental stress. Furthermore, as the simulation focused on caring for children, students experienced additional feelings of insecurity, despite being well prepared. Several students commented that the simulation had caused them to read more than usual and the simulation was not as scary as many had thought it would be. One student shared:

Even though you are stressed, you manage to think and stay fairly calm. So it’s a positive experience (FG5 P2).

Some students indicated that the stress they felt during the simulation made it difficult to manage the situation. A lack of knowledge of the situation and anxiety about managing other students kept them from taking the lead. Several of the students had planned which one of them should take the lead in advance, but this often did not work out due to unexpected events. However, the students felt they learned a lot about themselves, as well as how they appear as nurses.

It surprised me to watch the video and know how stressed you were in your head and then it didn’t show. And you still manage to do all you need to do. One feels that one manages to perform even if one is stressed (FG6 P2).

The students felt that fully experiencing physical stress (e.g. a higher pulse and increased sweating) helped them to better remember the skills learned in the simulation. They also felt that the situation was chaotic at times because everyone—or sometimes no one—took the lead, but that it was instructive in the debriefing to discuss management (e.g. what makes you a good leader, as well as how to help each other succeed and communicate effectively). One student reflected on the situation:

There was no one in charge of keeping control, holding the lead then. We did not communicate so well really. So, we learned a lot because of it (FG4 P6).

Although the video recording was stressful for some, others liked that the respondents were not sitting in the same room, but rather followed the simulation through streaming. However, during the scenario, the students forgot about the video recording and concentrated on the tasks, as described in the following quote:

When one is to perform something while others are watching, one gets very stressed and afraid to make mistakes and then you have to read and prepare yourselves so incredibly much in advance … But I felt that I was learning a lot about how to handle a situation, what can be done in that situation, and yes, I felt that I really got a lot out of it. Got cleaned up in thoughts (FG2 P5).

## Discussion

When choosing learning methods in academia, one must consider and relate to the learning objectives when planning activities. The Bachelor of Nursing programme focuses on both theory and practice. One method that can be used to combine these two areas of learning is healthcare simulation; however, it is expensive and time-consuming [9, 10]. This dilemma creates the necessity to justify why simulation as a learning method provides students with a more realistic approach to solving practical challenges than traditional teaching. When paediatric emergencies occur in the clinic, students are often not first in line to respond and thus do not gain sufficient experience to learn how to deal with these situations [32]. Theories of reflection, such as Schön’s reflection theory, describe how students who participate in simulations of clinical situations have the opportunity to make decisions and exercise critical thinking [25]. The gravity of the scenario prompted the students to concentrate on the tasks at hand and inspired them to discuss their actions afterwards. They expressed that the simulation gave them a better understanding of the physiological and communicative challenges in an emergency than training traditionally in lectures or being left to themselves in large groups to train with fellow students. This is in accordance with Akselbo et al. [32] and Cant and Cooper [33], who emphasised that students feel more secure, competent, and able to cope with real-life emergencies following simulation. Simulated practice of nursing assessment and patient management prior to students’ clinical nursing settings is known to be a strong educational method when used in conjunction with other methods of teaching [33].

In the simulated emergency, the student plays the role of nurse and is therefore responsible for making the right decisions and acting reasonably. In this situation, the students experienced both physical and mental stress. They noted that this experience allowed them to understand how a real-life emergency would feel. The quality of a student’s experience in tending to an emergency depends on the student’s clinical practice and the student tutor’s competence [34]. Therefore, the opportunity to be the one addressing an emergency situation in a controlled environment is an experience that students appreciate. The students’ experience of stress should be a driver of, rather than an obstacle to, learning. Furthermore, being able to discuss the simulation and highlight effective actions meant that the students experienced a broader and deeper understanding of the event. The students also recognised the gaps in their own knowledge and were able to discuss with the teacher how to obtain the knowledge they felt they were lacking. This finding is supported by another Norwegian study [32]. However, a systematic review [35] has shown that the effect of simulation training on students’ ability to think critically is difficult to measure. The inconsistent results obtained by various studies may be attributable to the use of different measurement instruments.

The debriefing was related to the steps of Kolb’s learning circle [12]. According to Kolb, learning occurs through change and adaptation of pre-existing knowledge. Kolb suggested that learning is an ongoing process, consisting of four stages as part of a cyclical process. In this study, the stages were as follows: In stage 1, the students described their initial reactions and their experiences during the scenario. In stage 2, the students reflected on what they had done well and what they could have done differently. In stage 3, the students discussed the theoretical and practical knowledge gained during the simulation. Finally, in stage 4, the students were asked how they thought they would act in a similar situation in the future, encouraging the adaptation of their newly acquired knowledge for future practice.

Schön’s theory may help to explain precisely what occurs during the processes of debriefing and reflection. He introduced the concept of the reflective practitioner, which distinguishes between the reflection that happens during the act and the critical reflection that occurs after the act [13]. When the students were in an unfamiliar situation that required certain skills, such as the simulated scenario in the present study, the actions appropriate to the scenario required knowledge not yet acquired by the students. The students were forced to rely on the competence and knowledge they had possessed prior to the start of the simulation. This led to what Schön termed ‘reflection-in-action’. During the debriefing that followed the simulation, in which the students reflected on the actions they took to solve the problem in the simulated scenario, further knowledge was developed. Schön termed this process reflection-on-action and claimed that such reflection enabled one to bridge the gap between theory and practice. The ability to reflect in action during simulation is a key factor in ensuring the best possible patient care in an emergency [13].

Taking leadership in emergencies requires the nurse to rapidly analyse a complex environment. The nurse must assess where and what sort of help is required and to communicate effectively to deliver that help [36]. The students in this study expressed high levels of stress, both before and during the simulation, due to low self-confidence from lack of knowledge and experience with emergencies. They expressed a desire for a greater number of simulation opportunities throughout their education, as they felt this learning method would help ease their stress and produce positive learning outcomes. Indeed, low self-confidence is associated with high levels of anxiety and delay in implementing expected actions, as well as more errors [37]. The competence gained through simulation (e.g. knowing what is going to happen and how) helps to raise confidence and reduce stress levels [38]. Repeated simulation experiences increase students’ self-confidence levels [37], and the more students work with critical thinking situations, the greater their ability will be to refine and build on their performance strategies [39]. Gaining the experience of an emergency in a controlled environment is important for feeling autonomous and improving confidence [40–42].

The university aspires to the delivery of competent nurses who are able to care for patients with acute, critical, and chronic illnesses and who have the ability to communicate and interact effectively with patients, relatives, and colleagues. Simulation as an active learning method is a well-documented tool for achieving these goals [43].

## Limitations

This study had both limitations and strengths. Despite the small sample size, the findings remain relevant. The students offered detailed descriptions of their experiences of simulation as a learning method. These descriptions improve our understanding of a number of important factors to be considered when planning future simulation training. One strength was that all the students participated in the study.

## Conclusion

The bachelor-level student nurses experienced simulation as a realistic and effective educational method for gaining knowledge in relation to the critical healthcare of children. The simulation increased participants’ preparedness, reduced their stress levels, and prompted the students to reflect on the scope of a nurse’s responsibility in an emergency situation. When students are offered practical experience in children’s wards, the use of simulation as a didactic method may help students develop sufficient competence to act appropriately and expediently in critical paediatric nursing settings.

## Supplementary information

**Additional file 1: Scenario 1.**

**Additional file 2: Scenario 2.**

## Data Availability

The datasets used and/or analysed during the current study are available from the corresponding author on reasonable request.
