# Supplementary material for: Simulation as a pedagogical learning method for critical paediatric nursing in Bachelor of Nursing programmes: a qualitative study
Source: Adv Simul (Lond). 2020 Sep 3;5:24. doi: 10.1186/s41077-020-00140-2 (PMC7499857; doi:10.1186/s41077-020-00140-2)
Supplement: Supplementary file 1 — Additional file 1: Scenario 1. [file 41077_2020_140_MOESM1_ESM.docx]

|  |  |
| --- | --- |
| **Additional file 1** |  |
| **Scenario** 1 | **6-year-old child with pyelonephritis and anaphylactic reaction** |
| **Prepared by** |  |
| **Study programme** | Bachelor of Nursing |
| **Subject/topic code** |  |
| **Course coordinator** |  |
| **Learning outcome from study plan** | The student is expected to have knowledge of: - nursing for patients with acute, critical, and chronic illness and suffering - communication and interaction with focus on acute, critical, and chronically ill patients and their relatives |
| **Learning outcome for the simulation exercise** | The student should:   1. Be able to use ABCDE and act in consideration of the situation 2. Be able to interact, lead, and communicate in emergencies using ISBAR |
| **Simulation Level** | 3 |
| **Expected knowledge before simulation** | Attended teaching: Introduction to the children's project. |
| **Recommended sources** | Grønseth and Markestad, 2017; Pediatric and Pediatric Nursing Chapters 5, 7, 17, and 19; Pediatric Early Warning Scores (PEWS) |
| **Roles and functions** | Simulating participants, respondents, facilitator, operator, extras, or stand-in |
| **Role description** | The three students are the nurses. The extra is the mother or father. The operator is the doctor communicating by phone and the voice of the 6-year-old child. |
| **Expected Progress** | After measuring PEWS, the child had cyanosis, SaO2 78, pulse 115, BT 75/40, respiration rate 55 with stridor, capillary refill 4 s. Called the doctor who ordered: stop antibiotics, administer adrenaline 0.01 mg/kg body weight (adrenaline 1 mg/mL; here the students should calculate the dose = 0.25 mL intramuscular), NaCl 0.9mg/mL 500 mL intravenous (students are expected to replace tubing sets), O_2_ on mask 10 l of flow. The doctor requests that the child is monitored and that the emergency trolley is brought to the room. The child reacts with weak cry asking for his mother. The mother seems afraid and worried. |
|  |  |
| **Name** | Lind, Emil |
| **Gender** | M |
| **Date of Birth** | 17.02.2012 |
| **Age** | 6 years old |
| **Weight** | 25 kg |
| **Height** | 115 cm |
| **Allergy** | Not known |
| **Medication** | Antibiotics: Cephalothin sodium 300 mg x 4/day intravenously |
| **Annet** | None |
| **History** | Previously healthy. Has now had pain in the stomach and back as well as vomiting. Fever 39 ºC for 2 days. Feeling slack and experiencing poor general condition. |
| **Current** | The patient arrived at the children's reception department at 08.00. Urine dipstix at the emergency room showed + ++ white blood cells and protein. Urine and blood culture taken. Blood samples showed CRP 120. Antibiotics administered: Cephalotin sodium 300 mg x 4 intravenously. First dose was given at 09.00. The child is transferred to a medical children's department. You arrive at work at 15.00. New dose of antibiotics has just started. Mother is calling, the child is pale, he itches, has urticaria, is breathing quickly, and seems lethargic. You measure PEWS. Monitoring equipment is available. |
|  |  |
| **Respondent tasks Group A** | Students will be able to use ABCDE, PEWS and act in consideration of the situation |
| **Gespondent tasks group B** | Students will be able to interact, lead, and communicate in emergencies using ISBAR |
| **Room for simulation** |  |
| **Room for transfer** |  |
| **Simulator** | SIM junior 2015 |
| **Preparation of mannequins and environment **** | Underwear, panties |
| **Available equipment ***** | Monitoring equipment, pulse oximeter, and BT cuff for 6 years.  On trolley: oxymask, adrenaline 1 mg/mL, saline for infusion, 1 mL syringes, intramuscular needle (blue or green), NaCl 0.9mg/mL 500 ml, IV tubes, antibiotics IV (note 100 mL infusion Keflin 300 mg), PEWS form and stethoscope. |
| **other** | Paper, pen, and telephone number of the doctor (operator) |
| **Rotation schedule** | 09.15-09.45 Briefing 09.45-10.00 Scenario 1 – Group 1, Unit X 10.00-10.45 Debriefing 10.50-11.00 Rebriefing 11.00-11.15 Scenario 2 – Group 1, Unit Y 11.15-12.00 Debriefing  12.00-12.45 Lunch  12.45-13.15 Briefing 13.15-13.30 Scenario 1 – Group 2, Unit X 13.30-14.15 Debriefing  14.20-14.30 Rebriefing 14.30-14.45 Scenario 2 - Group 2, Unit Y 14.45-15.30 Debriefing |
