# Supplementary material for: Simulation as a pedagogical learning method for critical paediatric nursing in Bachelor of Nursing programmes: a qualitative study
Source: Adv Simul (Lond). 2020 Sep 3;5:24. doi: 10.1186/s41077-020-00140-2 (PMC7499857; doi:10.1186/s41077-020-00140-2)
Supplement: Supplementary file 2 — Additional file 2: Scenario 2. [file 41077_2020_140_MOESM2_ESM.docx]

| **Additional file 2** |  |
| --- | --- |
| **Scenario 2** | **Infant with acute bronchiolite/Respiratory syncytial virus infection** |
| **Prepared by** |  |
| **Study programme** | Bachelor of Nursing |
| **Subject/topic code** |  |
| **Course coordinator** |  |
| **Learning outcome from study plan** | The student is expected to have knowledge of: (a) Nursing for patients with acute, critical, and chronic illness and suffering (b) Communication and interaction with focus on acute, critical, and chronically ill patients and their relatives |
| **Learning outcome for the simulation exercise** | The student should:   1. Be able to use ABCDE and act in consideration of the situation 2. Be able to interact, lead, and communicate in emergencies using ISBAR |
| **Simulation Level** | 3 |
| **Expected knowledge before simulation** | Attended teaching: Introduction to the children's project. |
| **Recommended sources** | Grønseth and Markestad, 2017; Pediatric and Pediatric Nursing Chapters 5, 7, 17, and 19; Pediatric Early Warning Scores (PEWS) |
| **Roles and functions** | Simulating participants, respondents, facilitator, operator, extras or stand-in |
| **Role Description** | The three students play the three nurses. The extra is the mother or father. The operator is a doctor communicating by phone. |
| **Expected Progress** | After measuring PEWS, the child is slack, has cyanosis, SaO_2_ 72, pulse 160, respiration rate 60 with stridor, capillary refill 4 s. Called the doctor who said she is busy and will arrive in 10 minutes. The doctor prescribes inhalation 2 mL sodium chloride 9 mg/mL. The child cries when given the inhalation. Mother seems desperate and afraid. The doctor (facilitator) comes in when the child has received inhalation. |
|  |  |
| **Name** | Lind, Ida |
| **Gender** | K |
| **Date of Birth** | 27.12.17 |
| **Age** | 5 weeks |
| **Weight** | 5 kg |
| **Height** | 55 cm |
| **Allergy** | Unknown |
| **Medication** | Antibiotics: Keflin 300 mg x 4/day intravenously |
| **Annet** | None |
| **History** | Has had a cold for two days, and is experiencing problems breastfeeding. Fever 38.5 ºC rectal at 12:00 – measured at home |
| **Current** | Mom brings the child to child emergency reception. After triage, medical supervision, and a nasopharynx test, the child is transferred to the Department for Child Infection. A sodium chloride inhalation was given in the emergency. Now the child has been transferred to the children’s infectious diseases ward. The child is being monitored. Now the mother calls for help. |
|  |  |
| **Respondent tasks group A** | Students will be able to use ABCDE and act in consideration of the situation |
| **Respondent tasks group B** | Students will be able to interact, lead, and communicate in emergencies using ISBAR |
| **Room for simulation** |  |
| **Room for transfer** |  |
| **Simulator** | SIM Newborn |
| **Preparation of mannequins and environment **** | Diaper, body, blanket |
| **Available equipment ***** | Monitoring equipment, pulse oximeter, O_2_ with mask and atomizing chamber, racemic adrenaline, saline, syringes, stethoscope |
| **other** | Paper, pen, and telephone number of the doctor (operator) |
| **Rotation schedule** | 09.15-09.45 Briefing 09.45-10.00 Scenario 1 – Group 1, Unit X 10.00-10.45 Debriefing 10.50-11.00 Rebriefing 11.00-11.15 Scenario 2 – Group 1, Unit Y 11.15-12.00 Debriefing  12.00-12.45 Lunch  12.45-13.15 Briefing 13.15-13.30 Scenario 1 – Group 2, Unit X 13.30-14.15 Debriefing  14.20-14.30 Rebriefing 14.30-14.45 Scenario 2 - Group 2, Unit Y 14.45-15.30 Debriefing |
